# The relationship of dental students’ characteristics to social support, psychosocial factors, lifestyle, and quality of life

**DOI:** 10.1080/21642850.2022.2102017

**Published:** 2022-07-22

**Authors:** Andréa Neiva da Silva, Deison Alencar Lucietto, Maria Victória da Silva Bastos, Thainá Queiroz do Nascimento, Mario Vianna Vettore

**Affiliations:** a Department of Health and Society, Institute of Collective Health, Fluminense Federal University (UFF), Niterói, Brazil; b Faculty of Dentistry, Fluminense Federal University (UFF), Niterói, Brazil; c Department of Health and Nursing Sciences, University of Agder (UiA), Kristiansand, Norway

**Keywords:** Psychosocial factors, health risk behaviour, quality of life, dental students, sense of coherence, social support

## Abstract

**Objective:**

To examine the relationships between sociodemographic characteristics, student academic information, social support, sense of coherence, anxiety, lifestyle, and quality of life among dental students.

**Methods:**

A cross-sectional study among 233 dental students in Brazil. We captured data on sociodemographic and socioeconomic characteristics, social support through Social Support Appraisal, psychosocial factors (sense of coherence and anxiety based on SOC-13 and Depression, Anxiety and Stress Scale-21 – anxiety subscale, respectively), lifestyle as per individual Lifestyle Profile and quality of life based on VERAS-Q. Data was analysed through Structural Equation Modelling.

**Results:**

Greater social support, higher sense of coherence, lower anxiety, better lifestyle directly predicted better quality of life. Male gender, city of origin did not differ from the city of the campus, higher social support and greater sense of coherence were indirectly linked with better quality of life via better lifestyle. Lower academic semester and higher social support indirectly predicted better quality of life via lower anxiety.

**Conclusion:**

Social support, sense of coherence, anxiety, and lifestyle were relevant factors directly associated with dental student’s quality of life. Indirect pathways were observed between gender, moved home to attend dental course, academic semester, social support, sense of coherence, and quality of life.

## Introduction

University life involves a range of unique opportunities for undergraduate students, including experiencing new physical and social environments as well as developing new social relationships that can potentially influence their well-being and quality of life (Elani et al., 2014; Harris, Wilson, Highes, Knevel, & Radford, 2018). During this period, students need to adapt to deal with the new challenges and responsibilities, as well as academic demands and pressures encountered in the academic environment (Polychronopoulou & Divaris, 2009; Tosevski, Milovancevic, & Gajic, 2010). Therefore, the excitement and empowering feelings experienced by undergraduates are commonly accompanied by worries and concerns that may negatively impact the adoption of unhealthy behaviours and psychological health (Huang et al., 2011; Naidu, Adams, Simeon, & Persad, 2002).

The increase in academic overload, constant pressure to succeed, competition with peers, financial burden in some countries, and concerns about professional future are some of the stressors acknowledged by university students (Tosevski et al., 2010). This scenario seems worse among health sciences students as they are more likely to have higher levels of anxiety and depression than those attending non-healthcare-related subjects (Papadopoulou et al., 2021). Moreover, dental and medical students also report an increase in work overload, overextended at work, and worry propensity as they progress through the course (Naidu et al., 2002; Sanders & Lushington, 1999; Schmitter, Liedl, Beck, & Rammelsberg, 2008). Overall, the levels of stress are more pronounced among dental students when compared with other healthcare students (Schmitter et al., 2008) due to increased competition for grades (Elani et al., 2014; Naidu et al., 2002), greater workload (Alhajj et al., 2018; Elani et al., 2014; Polychronopoulou & Divaris, 2009), higher demand and requirements for manual skills for laboratorial activities and clinical practice, strained relationships with professors, and lack of leisure time (Alhajj et al., 2018; Elani et al., 2014).

The adoption of unhealthy habits among undergraduate dental students, including alcohol consumption, binge drinking, and use of illicit drugs has been a matter of concern (Huang et al., 2011; Puryer & Wignall, 2016; Saxena, Mani, Dwivedi, Ryali, & Timothy, 2019). It has been suggested the link between risk behaviours, stress, and anxiety among dental students, which is triggered by poor academic experiences (Fujita & Maki, 2018; Hudd et al., 2000; Saxena et al., 2019). Psychological health, health-related behaviours, and academic performance have been considered important predictors of quality of life in younger adults and undergraduate students (Krzepota, Biernat, & Florkiewicz, 2015; Shareef et al., 2015). Quality of life can be defined as ‘an individual’s perception of their position in life, in the context of the culture and value systems in which they live, and in relation to their goals, expectations, standards, and concerns’ (WHO, 1993).

The challenging situations experienced during university studies do not necessarily generate psychological distress among students. Some students can develop and use effective strategies to deal with the adversities during the academic period. The salutogenic approach is a useful theory to explain how individuals effectively manage and cope with adverse and stressful situations and remain healthy (Antonovsky, 1987). Personality traits, gender, socio-cultural features, and psychosocial factors may mitigate the effect of difficulties experienced by students throughout the undergraduate course (Muirhead & Locker, 2008). However, research on protective psychosocial factors that facilitate coping strategies, including sense of coherence, and social support amongst undergraduate dental students is scarce.

Sense of coherence is defined as a global orientation to view the external stimulus as structured, manageable, meaningful, and worthy of investment and engagement (Antonovsky, 1987). The greater the sense of coherence, the more effectively the individuals can overcome adversities and, consequently, maintain health and quality of life (Lindström & Eriksson, 2005). The general resources of resistance are also critical for individuals to effectively deal with stressors, and include financial resources, knowledge and intelligence, experience, self-esteem, healthy behaviours, coping strategy, and social support (Antonovsky, 1987). Social support is generally defined in terms of the availability of people who individuals trust, and on whom they can rely on and who will care for them (Berkman & Kawachi, 2000). Social support refers to the degree of available resources from interpersonal relationships that provide material and emotional support to individual during difficult situations (Due, Holstein, Lund, Modvig, & Avlund, 1999). Greater sense of coherence and social support were associated with lower levels of stress in undergraduate students (Chu, Khan, Jahn, & Kraemer, 2016; Elani et al., 2014; Laurence, Williams, & Eiland, 2009). Moreover, positive academic environment and healthy behaviours were inversely correlated with stress in this group (Elani et al., 2014; Laurence et al., 2009; Muirhead & Locker, 2008; Solis & Lotufo-Neto, 2019).

Previous studies have investigated the role of health-related behaviours, such as physical activity, internet addiction, and dietary patterns, on health-related quality of life among adolescents and university students (Krzepota et al., 2015; Shariati-Bafghi et al., 2021; Solis & Lotufo-Neto, 2019). In addition, stress, depression, anxiety, and burnout were associated with poor student’s quality of life (Machado et al., 2020; Solis & Lotufo-Neto, 2019). Better socioeconomic conditions and good perception of the educational environment were relevant predictors of quality of life amongst medical and nursing students (Aboshaiqah & Cruz, 2019; Solis & Lotufo-Neto, 2019; Tempski et al., 2015). However, there is a dearth of research on what factors influence dental student’s quality of life (Machado et al., 2020). The objective of this study was to test the associations between sociodemographic characteristics, student academic characteristics, social support, sense of coherence, anxiety, lifestyle features, and quality of life among undergraduate dental students.

## Methods

### Population and setting

A cross-sectional study involving undergraduate dental students from the Niteroi Campus of the Fluminense Federal University, Brazil was conducted.Fluminense Federal University is a large public university in the state of Rio de Janeiro that offers a nine-semester bachelor’s degree in dentistry. All undergraduate dental students regularly enrolled in any semester of the course in 2018 and those aged 18 years and older were eligible to participate.

### Sampling and data collection

Initially, academics involved in the classroom teaching activities at the dental school were informed about the aim of the study and their collaboration was requested. Then, the researchers agreed and scheduled specific dates for data collection with teachers of all semesters of the course in a routine day class. The complete list of enrolled dental students in each semester was previously obtained from the course coordination. All dental students who were in the classroom during the time of the data collection who fulfilled the inclusion criteria were invited to participate. At least three attempts of recruitment were made in each class when one or more students were missing in the class.

Recruitment and data collection was carried out at the classrooms at the dental school between August and October 2018 according to the following steps. First, two researchers informed the students the aim of the study and the data collection procedures. Any queries about the research were clarified. Second, the students were invited to participate in the study and those who agreed to participate signed the informed consent form. Third, detailed and standardized instructions on how to answer the questionnaire was provided by the research assistants. Finally, a self-completed questionnaire in Portuguese language was used to obtain information on demographics, socioeconomic factors, student academic characteristics, social support, psychosocial factors, lifestyle, and quality of life. The scales used to assess social support, psychosocial factors, lifestyle, and quality of life were previously validated for the Brazilian population.

### Response rate and study power

Overall, there were 258 undergraduate students enrolled at the dental school at the Fluminense Federal University in 2018. Of them, 233 composed the studied sample (response rate was 90.3%). Twelve students were excluded because they were younger 18 years old, one student refused to participate, and further 12 students were excluded due to missing data in one or more variables.

The final sample size of 233 participants would lend a power of 80% to detect statistically significant effects size of at least 0.24 for structural equation modelling with four latent variables and nine observed variables, and 5% level of significance (Westland, 2010). The study power for structural equation modelling was calculated using the statistical calculator available on https://www.danielsoper.com/statcalc.

### Theoretical model

**Figure 1.**
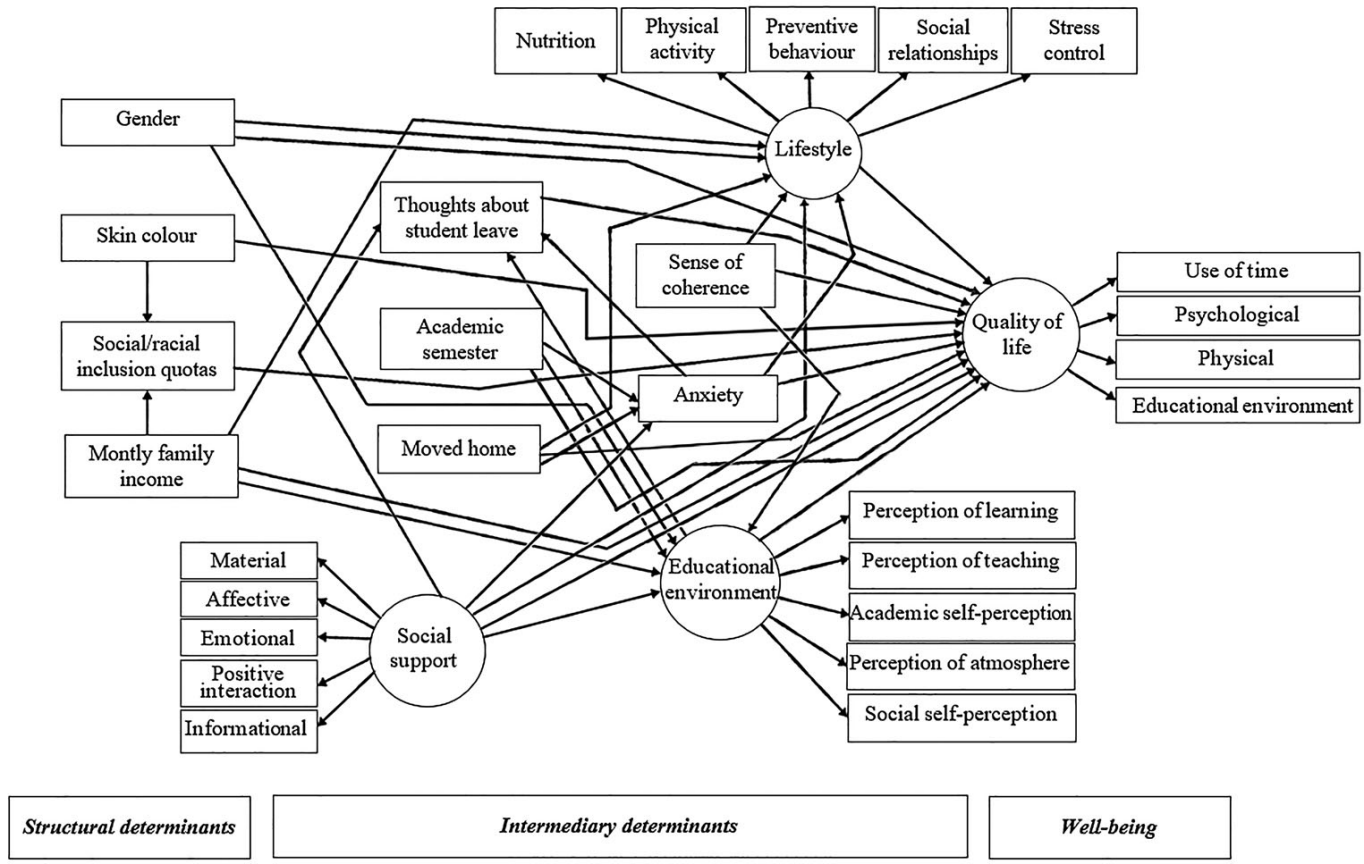
Full theoretical model on the relationships between sociodemographic characteristics, student academic characteristics, social support, psychosocial factors, lifestyle and quality of life in dental students according to the WHO conceptual framework of social determinants of health and well-being.

The adapted version of the World Health Organization conceptual framework of social determinants of health and well-being was used in the present study (WHO, 2010) (Figure 1). It was anticipated that structural determinants, including demographics and poor socioeconomic characteristics would predict intermediary determinants, including psychosocial factors, student academic characteristics, and worse lifestyle. It was also expected that the aforementioned structural and intermediary determinants would predict the worse quality of life. In addition, the intermediary determinants would mediate the relationship between structural determinants and quality of life.

### Variables

All scales used in this study were previously cross-culturally adapted for Brazilian adults and showed adequate psychometric properties in studies among Brazilian medical or dental students.

### Demographics and socioeconomic characteristics

Demographic data included age, gender (male/female), skin colour and whether the city of origin differs from the city of the campus. Undergraduate skin colour was a self-reported measure according to the classification established by the Brazilian Institute of Geography and Statistics (white, yellow, indigenous, brown, and black) (Travassos & Williams, 2004). Socioeconomic characteristics were monthly family income recorded in Brazilian Minimum Wages (BMWs) (<3 BMW, 3–6 BMW, > 6–10 BMW, >10 BMW) and if the participant was admitted at the university through social/racial inclusion quotas (no/yes). One BMW corresponded to U$ 235.53 in the study period (Exchange rates, n.d.).

### Student academic characteristics

Academic characteristics of the students were thoughts on applying for student leave, current academic semester, whether the student’s city of origin differs from the city of the campus (no/yes) and student’s perception of the educational environment. Thoughts on applying for student leave were assessed according to the question: ‘Have you been thinking in applying for student leave?’ (no/yes). Current academic semester was based on the academic semester they were regularly enrolled according to the course coordination, ranging from 1 to 9.

The Dundee Ready Educational Environment Measure (DREEM) questionnaire was used to evaluate dental student’s perceptions of the educational environment (De Oliveira Filho, Vieira, & Schönhorst, 2005; Roff et al., 1997). The DREEM is composed of 50 items followed by a 5-point Likert scale ranging from ‘0 = strongly disagree’ to ‘4 = strongly agree’. Student’s perception of the educational environment was a latent variable using the five domains of the DREEM as indicators: ‘students’ perception of learning’, ‘students’ perception of teachers’, ‘students’ academic self-perception’, ‘students’ perception of atmosphere’, and ‘students’ social self-perception’. Higher scores indicate a more positive evaluation of the educational environment. DREEM was considered a suitable instrument for measuring the perception of educational environment in undergraduate medical students (Soemantri, Herrera, & Riquelme, 2010). The Cronbach’s alpha of the DREEM scale in studies carried out with medical students in Brazil ranged from 0.76 to 0.94 (Costa, Costa, & Pereira, 2021; Miguel, Tempski, Kobayasi, Mayer, & Martins, 2021).

### Social support

The social support scale was used to assess perceived social support (Chor, Griep, Lopes, & Faerstein, 2001; Sherbourne & Stewart, 1991). The questionnaire consisted of 19 items, comprising 5 functional dimensions of social support: material; affective; emotional; positive social interaction, and informational. The following response options were used: never, rarely, sometimes, almost always, or always (Chor et al., 2001; Sherbourne & Stewart, 1991). Social support was a latent variable using the scores of each dimension as indicators. Higher scores on social support scale indicate greater perception of social support. The validation study of the social support scale in Brazilian adults reported a Cronbach’s alpha coefficient of at least 0.83 across the scale dimensions (Griep, Chor, Faerstein, Werneck, & Lopes, 2005). Previous research using the social support scale among Brazilian university students showed excellent internal consistency in all domains: material (*α* = 0.85), affective (*α* = 0.88), emotional (*α* = 0.92), positive social interaction (*α* = 0.94), and information (*α* = 0.92) (Silva et al., 2021).

### Psychosocial factors

Sense of coherence was measured through the cross-culturally adapted Brazilian short version of the Sense of Coherence scale (SOC-13) (Antonovsky, 1987; Bonanato et al., 2009). The scale consists of 13 items that are answered on a five-point Likert scale. Explanations for intermediate answers and semantic limits of extreme answers are provided. The sense of coherence score is obtained by adding up the scores sum of the 13 items. The nine items related to negative SOC are reversed before calculating the total SOC score. The higher the score, the stronger the sense of coherence. Previous studies showed that SOC-13 presented satisfactory internal consistency when used among Brazilian adults (*α* = 0.80) (Davoglio et al., 2016) and adolescents (*α* = 0.67) (Silva et al., 2020). Cronbach’s alpha coefficient of the SOC-13 was 0.94 among undergraduate dental students in Brazil (Silva et al., 2021).

Anxiety was assessed based on perceived symptoms of anxiety using the seven items of the anxiety subscale of Depression, Anxiety, and Stress Scale (DASS-21) (Lovibond & Lovibond, 1995; Vignola & Tucci, 2014). The anxiety subscale of the DASS-21 is a four-point Likert scale, ranging from 0 = ‘strongly disagree’ to 3 = ‘totally agree’. Anxiety score is obtained by summing the items 2, 4, 7, 9, 15, 19, and 20 of the DASS-21. The Cronbach’s alpha of the anxiety subscale of the DASS-21 of the validation study for the Brazilian population was 0.92, supporting the reliability of the subscale (Vignola & Tucci, 2014). The Cronbach’s alpha of the DASS-21 anxiety subscale among Brazilian university students, including medical and dental students, ranged from 0.87 to 0.91 (Lopes & Nihei, 2021; Moutinho, Lucchetti, Ezequiel, & Lucchetti, 2019; Silva et al., 2021).

### Lifestyle

Student’s lifestyle was assessed through the Individual Lifestyle Profile (ILP) scale (Martins, Marôco, Barros, & Campos, 2020). The instrument involves five components: nutrition, physical activity, preventive behaviour, social relationships, and stress control (Martins et al., 2020). The scale consists of 15 items answered on a four-point Likert scale: ‘0 = absolutely not’, ‘1 = sometimes’, ‘2 = often’, and ‘3 = always’. The codes were summed to obtain the score of each component of the ILP scale. Greater scores of ILP scale indicate a healthier lifestyle. Lifestyle was a latent variable using the scores of each component as indicators. The ILP scale Cronbach’s alpha was 0.71 in the validation study for the Brazilian population (Nahas, Barros, & Francalacci, 2000). Cronbach’s alpha was 0.77 among Brazilian university students (Hernandez et al., 2007).

### Quality of life

Quality of life was assessed using the Life of the Health Student and Health Resident questionnaire (VERAS-Q). The scale was originally developed and validated to assess the quality of life of medical students with the internal consistency of 0.78 (Martins et al., 2020). The 45 items of the VERAS-Q include conceptual aspects and determinants of quality of life of health sciences students. VERAS-Q is a five-point Likert scale ranging from ‘1 = strongly disagree’ to ‘5 = totally agree’, divided into four domains: use of time, psychological, physical, and educational environment. The scores of the subscales are calculated by summing the items of each subscale (Martins et al., 2020). Higher scores denote better quality of life. Quality of life was a latent variable using the scores of the five domains as indicators. The VERAS-Q Cronbach’s alpha of previous studies among Brazilian medical students ranged from 0.70–0.91 (Miguel et al., 2021), (Barros, Menezes, & Lins, 2019; Paro et al., 2019; Tempski, Perotta, Pose, & Vieira, 2009).

### Reliability of the instruments

Omega coefficient was used to assess the internal consistency of the instruments DREEM, social support scale, SOC-13, DASS-21 anxiety subscale, ILP scale, and VERAS-Q. The Omega coefficients were: DREEM = 0.928, social support scale = 0.951, SOC-13 = 0.829, DASS-21 anxiety subscale = 0.846, ILP scale = 0.685, and VERAS-Q = 0.916.

### Data analysis

The distribution of the continuous and categorical variables was presented through means and proportions and the respective 95% confidence intervals (CIs) for the total sample and according to gender groups. Categorical variables were compared against gender by using Pearson’s chi-square test. The comparison of the continuous variables between gender groups was checked by *t*-test and Mann–Whitney test. Shapiro–Wilk test was used to assess whether continuous variables were normally distributed. The hypothesised measurement model was tested using confirmatory factor analysis (CFA) to assess the loading factors and statistical significance between the latent variables and their observed measures (indicators). The dichotomous variables were dummy-coded to reflect the categorical nature of the variables before including them in the statistical modelling.

Structural equation modelling (SEM) tested the direct and indirect relationships between the observed and latent variables according to the WHO conceptual framework of social determinants of health and well-being (WHO, 2010) (Figure 1). The standardized total, direct, and indirect effects were estimated using the Maximum likelihood estimation method. Total indirect effects represent the sum of one or more specific paths, which represents the sum of the direct link from one variable to another and the indirect effects where the link is mediated by other variables. The standard errors and 95% CI were used to assess mediation by analysing the statistical significance of indirect effects (Tempski et al., 2009). After estimating the full model (see Appendix Table A), non-significant direct paths were removed, and a statistically parsimonious model was re-estimated. The full model and the parsimonious model were compared using the Likelihood-ratio test. The standardized root mean square residual (SRMR) ≤ 0.08, comparative fit indices (CFI) ≥ 0.90, and Coefficient of Determination (CD) ≥ 0.90 were adopted to assess the adequacy of the measurement and structural models (MacKinnon, Lockwood, Hoffman, West, & Sheets, 2002). The significance level established for all analyses was 5% (*p* ≤ 0.05). All analyses were performed using statistical software STATA 16.0 (StataCorp, College Station, TX, USA).

### Ethical considerations

The present study was conducted in accordance with the Declaration of Helsinki. The research protocol was approved by the Ethics Research Committee of the Faculty of Medicine/University Hospital Antônio Pedro of the Fluminense Federal University (Protocol no. 2.721.482). All participants of the study provided signed informed consent before data collection.

## Results

The studied sample included 233 participants with a mean age of 22.2 years old (SD = 3.7). Most participants were females (82.8%), had white skin colour (58.4%), and monthly family income between 3 and 6 BMWs (33.9%%). Nearly 14% of the university students reported thoughts on applying for student leave and more than half of them were from the same city of the campus. Women reported lower income than men. Educational environment, sense of coherence and quality of life scores were significantly higher in men than in women. Anxiety scores were greater in women compared with men (Table 1).

**Table 1.** Sociodemographic characteristics, student academic characteristics, educational environment, social support, psychosocial factors, lifestyle, and quality of life, according to gender groups.

	*Study sample*		*Female participants*		*Male participants*		
Variable	*N* = 233	Mean (95% CI)/% (95% CI)	*N* = 193	Mean (95% CI)/% (95% CI)	*N* = 40	Mean (95% CI)/% (95% CI)	*P*
Demographics and socioeconomic characteristics							
Age, mean	233	22.2 (21.7–22.7)	193	23.7 (21.5–25.8)	40	21.9 (21.6–22.3)	0.670^a^
Skin colour, %							0.206^b^
White	136	58.4 (51.9–64.6)	110	57.0 (49.9–63.8)	26	65.0 (49.1–78.1)	
Black	17	7.3 (4.6–11.5)	12	6.2 (3.6–10.7)	5	12.5 (5.3–26.8)	
Brown	70	30.0 (24.5–36.3)	63	32.6 (26.4–39.6)	7	17.5 (8.5–32.5)	
Yellow	4	1.7 (0.6–4.5)	4	2.1 (0.8–3.3)	0	–	
Indigenous	2	0.9 (0.2–3.4)	1	0.6 (0.1–3.6)	1	2.5 (0.4–15.9)	
Did not declare	4	1.7 (0.6–4.5)	3	1.5 (0.5–4.7)	1	2.5 (0.4–15.9)	
Monthly family income, %							0.034^b^
< 3 BMW	56	24.0 (19.0–30.0)	49	25.4 (19.7–32.0)	7	17.5 (8.5–32.5)	
3–6 BMW	79	33.9 (28.1–40.3)	67	34.7 (28.3–41.7)	11	27.5 (15.9–43.3)	
> 6–10 BMW	53	22.8 (17.8–28.6)	46	23.8 (18.3–30.4)	7	17.5 (8.6–32.5)	
>10 BMW	45	19.3 (14.7–24.9)	31	16.6 (11.1–21.4)	15	37.5 (24.0–53.3)	
Social/racial inclusion quotas, %							0.679^b^
No	135	57.9 (51.5–64.1)	113	58.5 (51.4–65.3)	22	55.0 (39.5–69.6)	
Yes	98	42.6 (35.9–48.5)	80	41.5 (34.7–48.6)	18	45.0 (30.4–60.5)	
Student academic characteristics							
Thoughts on applying for student leave, %							0.803^b^
No	201	86.3 (81.2–90.1)	166	86.0 (80.3–90.2)	35	87.5 (73.2–94.7)	
Yes	32	13.7 (9.9–18.8)	27	14.0 (9.8–19.7)	5	12.5 (5.3–2.7)	
City of origin differs from the city of the campus, %							0.779^b^
No	106	45.5 (39.2–52.0)	87	45.1 (38.2–52.2)	19	47.5 (32.7–62.8)	
Yes	127	54.5 (48.0–60.8)	106	54.9 (47.8–61.8)	21	52.5 (37.2–67.4)	
Current academic semester, %							0.745^b^
1–3	87	37.3 (31.3–43.8)	74	38.3 (31.7–45.4)	13	32.5 (19.9–48.4)	
4–5	88	37.8 (31.7–44.2)	71	36.8 (30.3–43.9)	17	42.5 (28.2–58.1)	
6–9	58	24.9 (19.7–30.9)	48	24.9 (19.3–31.5)	10	25.0 (14.0–40.6)	
Educational environment (DREEM), mean	233	143.8 (60.0–227.5)	193	99.6 (96.1–103.1)	40	109.5 (102.8–116.1)	0.022^c^
Social support (SSA), mean	233	81.4 (79.4–83.4)	193	81.3 (79.1–83.5)	40	81.9 (77.2–86.6)	0.226^a^
Psychosocial factors							
Sense of coherence (SOC-13), mean	233	40.3 (39.6–41.2)	193	39.8 (38.9–40.8)	40	42.5 (40.0–44.9)	0.033^c^
Anxiety (DASS-21), mean	233	13.6 (12.2–15.0)	193	14.7 (13.2–16.2)	40	8.4 (5.3–11.4)	<0.001^a^
Lifestyle (ILP), mean	233	21.0 (20.1–21.8)	193	20.6 (19.7–21.6)	40	22.5 (20.3–24.6)	0.124^c^
Quality of life (VERAS-Q)	233		193		40		
Total score, mean		123.3 (120.3–126.2)		120.8 (117.7–123.9)		135.2 (127.8–142.6)	0.001^a^
Use of time, mean		26.8 (25.7–27.8)		25.8 (24.7–26.9)		31.3 (28.8–33.8)	<0.001^a^
Psychological, mean		32.5 (31.6–33.5)		31.9 (30.9–33.0)		35.5 (32.8–38.1)	0.008^a^
Physical, mean		20.0 (19.3–20.6)		19.5 (18.8–20.2)		22.3 (20.7–23.9)	0.002^a^
Educational environment, mean		44.0 (13.1–44.9)		43.6 (42.6–44.5)		46.2 (43.9–48.5)	0.032^c^

BMW, Brazilian minimal wage (1BMW = 226$).

^a^Mann–Whitney test.

^b^Pearson’s chi-square test.

^c^*t*-test.

CFA supported the hypothesised measurement model with the values: SRMR = 0.060, CFI = 0.975 and CD = 1.00. Similarly, SEM supported the hypothesised full model and the parsimonious model. The fit indices of the former were SRMR = 0.068, CFI = 0.955 and CD = 1.00. The fit indices of the parsimonious model were SRMR = 0.071, CFI = 0.936 and CD = 1.00. The regression weights showed that monthly family income was not significantly associated with any variables in the model. Thus this variable and non-significant direct paths were removed to enhance statistical parsimony.

**Figure 2.**
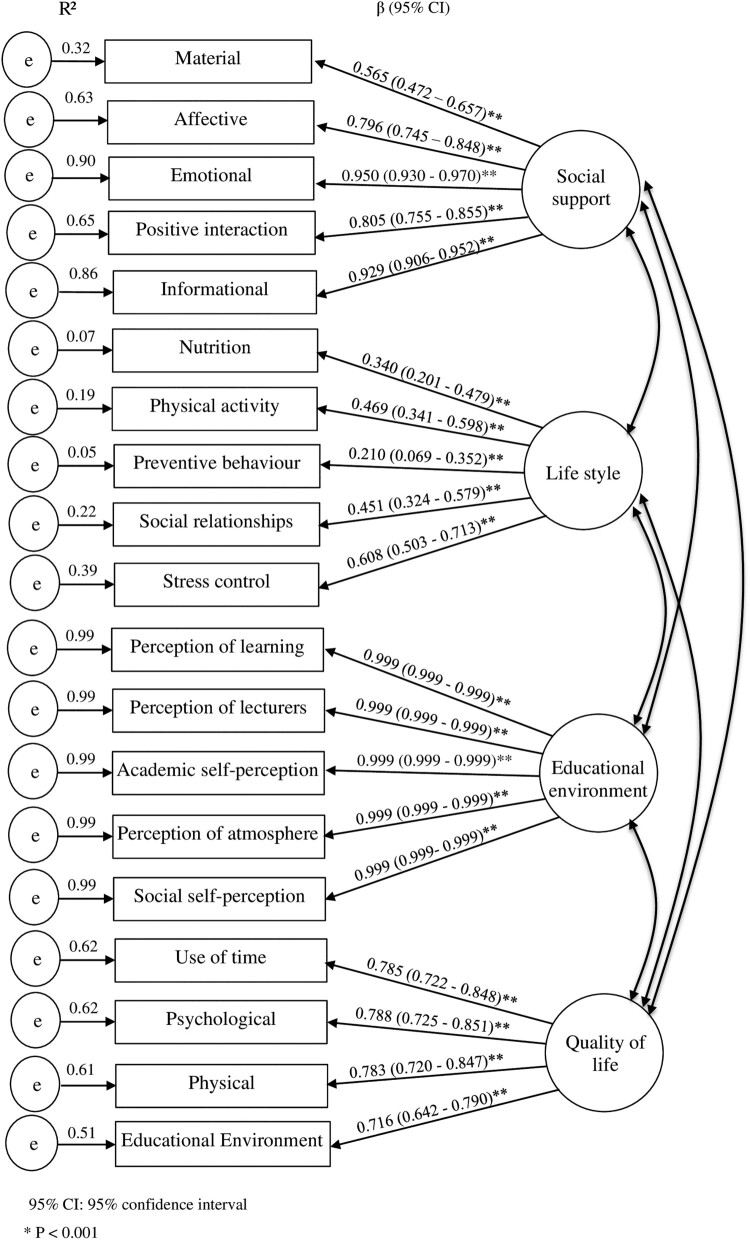
Confirmatory factor analysis of the four latent variables (measurement model).

The confirmatory factor analysis (CFA) assessing the measurement model for the four latent variables is reported in Figure 2. The latent variables ‘social support’, ‘lifestyle’, ‘student’s perception of the educational environment’ and ‘Quality of life’ were confirmed using the respective indicators.

**Figure 3.**
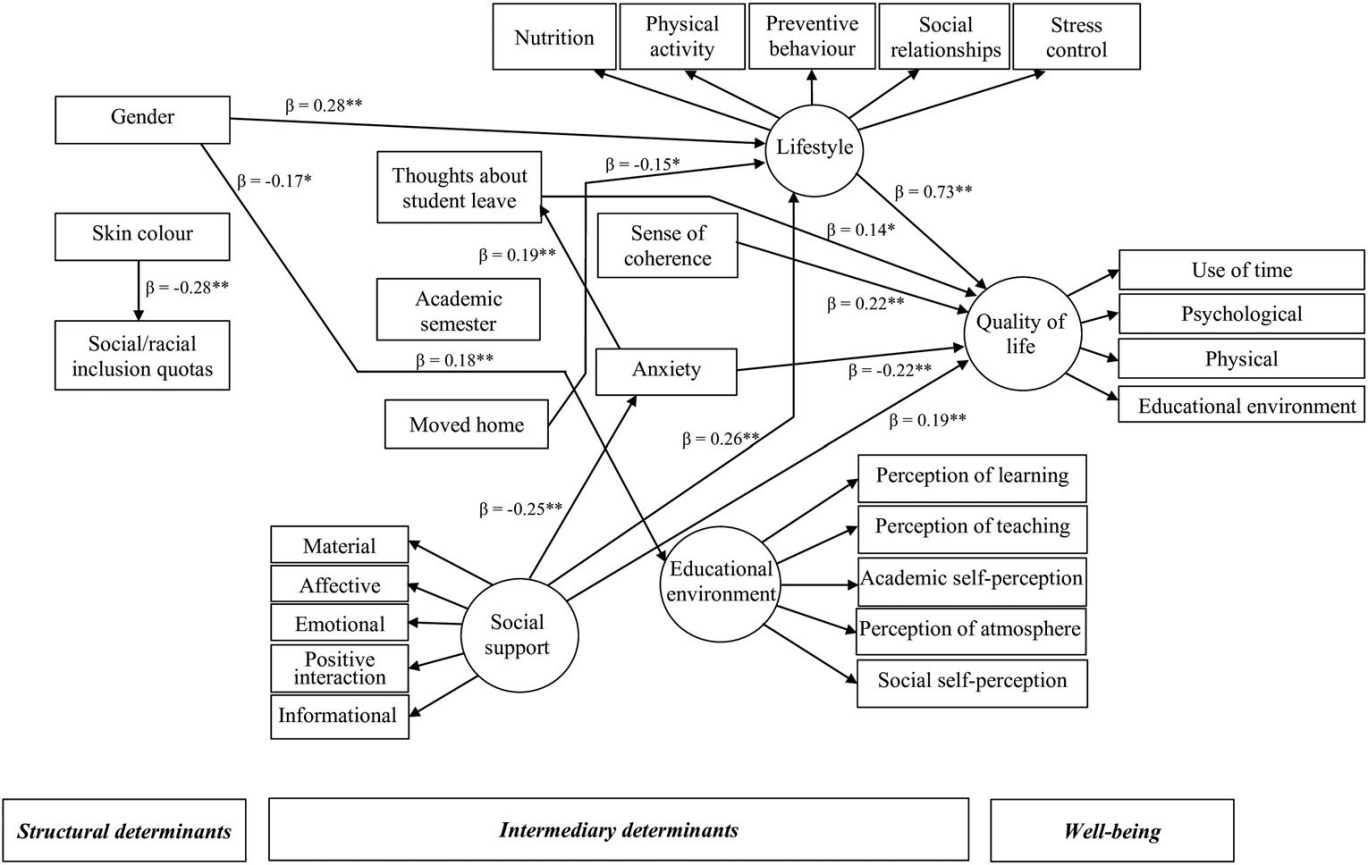
Parsimonious model of the relationships between sociodemographic characteristics, student academic characteristics, social support, psychosocial factors, lifestyle, and quality of life. All figures are standardised beta coefficients: *P < 0.05, **P < 0.01.

The direct relationships of the parsimonious model are summarized in Figure 3. Greater social support (*β* = 0.19, *p* = 0.001), no thoughts about student leave (*β* = −0.14, *p* = 0.032), higher sense of coherence (*β* = 0.22, *p* = 0.005), lower anxiety (*β* = −0.22, *p* < 0.001), and healthier lifestyle (*β* = 0.73, *p* < 0.001) were directly linked with better quality of life. Female gender directly predicted worse lifestyle (*β* = −0.23, *p* = 0.001) and poor perception of educational environment (*β* = −0.17, *p* = 0.014). Skin colour was directly associated with admission at the university through social/racial inclusion quotas (*β* = 0.28, *p* < 0.001). Higher academic semester was directly associated with higher anxiety (*β* = 0.18, *p* = 0.003). City of origin different from the city of the campus was directly linked to worse lifestyle (*β* = −0.15, *p* = 0.034). Higher social support was directly associated with low anxiety (*β* = −0.25, *p* < 0.001) and better lifestyle (*β* = 0.26, *p* = 0.004). Greater sense of coherence directly predicted better lifestyle (*β* = 0.34, *p* < 0.001). Higher anxiety was directly related to students’ thoughts on applying for student leave (*β* = 0.19, *p* = 0.003).

Significant indirect relationships of gender (*β* = 0.17), academic semester (*β* = −0.04, *p* = 0.001), city of origin different from the city of the campus (*β* = −0.11, *p* = 0.049), social support (*β* = 0.24, *p* = 0.001), sense of coherence (*β* = 0.25, *p* = 0.007), and anxiety (*β* = 0.03, *p* = 0.04) with quality of life were identified. Male gender, city of origin not different from the city of the campus, higher social support and greater sense of coherence indirectly predicted quality of life via better lifestyle. Moreover, lower academic semester and higher social support were indirect predictors of better quality of life via lower anxiety. Greater anxiety was indirectly linked with poor quality of life via students’ thoughts on applying for student leave.

## Discussion

The herein presented study highlighted the complex relationships between sociodemographic characteristics, student academic characteristics, social support, psychosocial factors, lifestyle, and quality of life in undergraduate dental students. Gender was the only structural determinant associated with quality of life in this study. Recent evidence has shown that female gender and low socioeconomic background were meaningful predictors of poor quality of life among medical and nursing students (Aboshaiqah & Cruz, 2019; Solis & Lotufo-Neto, 2019). The mediation effect of lifestyle on the link between gender and quality of life is supported by previous research showing that female university students smoked less and were less physically active than male students (Fujita & Maki, 2018; Hu & Bentler, 1999). In contrast, the latter group reported a healthier diet than females (Hu & Bentler, 1999; Souza, José, & Barbosa, 2013). The influence of gender on lifestyle and quality of life might also be related to the worse stress control by female students (Alhajj et al., 2018; Ansari, Stock, & Mikolajczyk, 2012; Basudan, Binanzan, & Alhassan, 2017; Hudd et al., 2000).

Student academic characteristics, including higher current academic semester and moving to another city to study were indirectly linked with poor quality of life. Previous research has shown that years of study and distance from hometown were negatively associated with quality of life in medical students through anxiety and lifestyle (Harris et al., 2018). The demanding requirements of clinical training and the development of complex communicating skills to deal with patients are important sources of stress among dental students that may explain the decline of student quality of life (Alhajj et al., 2018; Elani et al., 2014; Gambetta-Tessini, Mariño, Morgan, Evans, & Anderson, 2013; Hayes, Hoover, Karunanayake, & Uswak, 2017; Naidu et al., 2002; Sanders & Lushington, 1999). In addition, undergraduate dental students in the final year of study are more likely to have greater anxiety and worse quality of life due to the proximity of entering in a saturated labour market in a developing country. The current dentist/population ratio in Brazil is 1 dentist/735 inhabitants, which is two times higher than the WHO recommendation (Paro & Bittencourt, 2013). Furthermore, the number of dental schools in Brazil increased 87% between 2015 and 2019 (Martin et al., 2018). The lack of association between student’s perception of the educational environment and quality of life was an unexpected finding. Possibly, psychosocial factors and behaviours were more meaningful determinants of student’s quality of life in our sample than the perception of the educational environment.

Social support and psychosocial factors were directly linked with student quality of life. This finding agrees with other studies revealing the importance of social bonding and ties on the quality of life in university students (Westland, 2010). The protective effect of social support on quality of life through buffering the symptoms of anxiety is reinforced by previous (Hayes et al., 2017; Laurence et al., 2009; Muirhead & Locker, 2008; Tempski et al., 2009). In addition, peer social support was the main contributor to healthy behaviours in health-related students (Wang, Koenig, Ma, & Shohaib, 2016). The impact of the high demand for dental training to acquire excellence in clinical, communication, and interpersonal skills may negatively influence psychological health. Psychological suffering of dental students is also related to academic factors (e.g. examinations and workload), peer pressure, and personal issues (Alhajj et al., 2018; Elani et al., 2014; Naidu et al., 2002; Sanders & Lushington, 1999). Moreover, high levels of stress were related to dental student physical health, such as number of times sick, loss of appetite, and sleeping and digestive problems (Elani et al., 2014).

Sense of coherence was associated with dental student’s quality of life via direct and indirect mechanisms. Sense of coherence was a significant predictor of quality of life among university students (Liu et al., 2020). The direct mechanism maybe because high sense of coherence enables students to understand the difficulties during academic training as challenges to be faced through the management of resources (material and symbolic) that are at their disposal (Antonovsky, 1987). This individual way of thinking, feeling, and acting with self-confidence generates a positive impact on student quality of life. Previous evidence on the relationship of sense of coherence with anxiety and lifestyle support the indirect effect of sense of coherence on quality of life via these mediators (Hayes et al., 2017; Kleiveland, Natvig, & Jepsen, 2015). There is sound evidence that higher sense of coherence is associated with lifestyle and healthy behaviours. Dental students with a higher sense of coherence are less likely to smoke (Mato & Tsukasaki, 2019) and to pay more attention to health and nutrition (Chu et al., 2016). Individuals engaging in a health-promoting lifestyle remain healthy and functional which contribute to a positive quality of life (Peker, Bermek, & Uysal, 2012). Smoking habits, alcohol, and other substances abuse may reflect the harmful outcomes of distress in many dental students (Elani et al., 2014; Hudd et al., 2000; Saxena et al., 2019).

Few studies have evaluated the possible influence of lifestyle on undergraduate student quality of life. According to our findings, lifestyle was the strongest direct predictor of student quality of life. Moreover, lifestyle was the main mediator of the relationship between other predictors and quality of life. Better quality of life among highly active respondents was observed among elderly university students around 60 years of age (Krzepota et al., 2015). In addition, healthy diet was associated with better quality of life in adolescents (Shariati-Bafghi et al., 2021). The mediation effect of lifestyle is also supported by previous evidence revealing that student’s attitudes, decision-making, and behaviours were affected by gender, whether the student was living at parental home, social support and sense of coherence (Nahas et al., 2000; Puryer & Wignall, 2016; Wang et al., 2016). In this study, not living with their families was associated with worse lifestyle, which in turn resulted in poor student quality of life. Undergraduate students from four European countries indicated that those living in parental home consumed more fruits and vegetables than those residing outside of their family home (Souza et al., 2013). Most university students who left their hometowns to study face additional challenges to have healthy behaviours as the new environment may play a role (Eriksson et al., 2010). In these cases, students dedicate less time to physical activity, relaxation, and leisure due to domestic tasks, which may impair their quality of life.

As far as the authors are aware, this is the first comprehensive research that has simultaneously tested the association of relevant structural and intermediary determinants with quality of life amongst dental students. However, the present study has some limitations. The studied sample was composed of dental students recruited in one public dental school in Brazil. Therefore, the present findings should not be extrapolated to undergraduates attending other subjects and those from different social and cultural backgrounds. The sample is predominantly composed of women (82.8%), which may impose restrictions on the generalization of our results. This finding reflects the greater proportion of women among dental undergraduate students in Brazil (≈75% of women) (Jamali et al., 2013). However, the observed relationships between variables assessed through structural equation modelling must be considered valid since gender was included in the parsimonious model. Structural equation modelling is considered a robust statistical method to test complex theoretical models and to test multiple relationships. Though, the present study might be subject to multiple comparisons problem, which may occur when a large number of relationships are tested simultaneously as it is more likely to find significant associations between variables as the number of hypothetical links proposed in the theoretical model increases. The cross-sectional design imposes restrictions about causal interpretations on the observed associations, and any observed relationships must rely on assumptions proposed by the theoretical model. Future longitudinal studies are important to confirm the possible causal links of relationships between variables identified in this study.

The present findings must be interpreted considering the socio-cultural aspects of the study population. The study was carried out in public university and included students from different socioeconomic background. Most of the study participants reported a monthly income of up to 6 BMW (57.9%) and 42.6% of the university students were admitted to the university through social/racial inclusion quotas. Thus protective psychosocial factors seem to exert an important influence on the quality of life of dental students from different socioeconomic background as observed in the present study. In addition, the high student’s financial costs for dental training in Brazil, which is related to the purchase of dental instruments, special clothes, and personal protection equipment for clinical and laboratory training. These financial expenses may represent a potential source of anxiety, especially among low-socioeconomic status dental students who participated in the study. It is also important to highlight that Brazilian dental students with low socioeconomic status tend to adopt unhealthy behaviours since they need to reconcile their academic activities with work, resulting in less time available for leisure, self-care, and physical activity.

The development of pedagogical activities that reinforce the cognitive, behavioural, and motivational capabilities of undergraduate students can contribute to shape a strong sense of coherence. In addition, there is a need to invest in a healthier, friendlier, and more welcoming academic environment in Brazilian universities. The student’s perception of social support from colleagues and teachers represents an important general resource of resistance that contributes to increase student’s sense of coherence and enhance their quality of life. These strategies can also contribute to reduce anxiety among university students, especially when they come from different sociocultural contexts, such as in public universities where the study was conducted. Dental training policies must consider the university as an institution that promotes health, care, and quality of life of students in addition to offer training for dental practice. Therefore, investments are needed in health promotion programs to encourage healthy eating habits, physical exercise, and psychological support to mitigate the effects of stress and anxiety during the course. Investing in dental training allied to strategies aiming to enhance the quality of life of future dentists can contribute to improving the quality of dental care.

## Conclusion

The present findings demonstrated that higher social support, greater sense of coherence, lower anxiety, and healthier lifestyle directly predicted better quality of life in dental students. Our data indicate the importance of the structural and intermediary determinants on the quality of life. In addition, anxiety mediated the relationship between current academic semester and social support with quality of life, whereas lifestyle mediated the relationship of gender, whether the student moved to another city to study, social support, and sense of coherence with quality of life. These findings highlight relevant pathways by which gender, student academic characteristics, social support, and sense of coherence influence the quality of life in undergraduate dental students.

## Data Availability

The data that support the findings of this study are available on request from the corresponding author. The data are not publicly available due to privacy and ethical restrictions. The eligible participants of the present study were all dental students aged 18 years and older from one university in Brazil – Fluminense Federal University. Since this information was disclosed in the manuscript, we would like to consider making the data available only upon request.

## Appendix Table A. Standardized effects of the full structural equation model.

**Table:**

	Quality of life		
Variables	β	95%CI	P
Gender	−0.03	−0.16/0.11	0.708
Skin colour	−0.05	−0.15/0.06	0.381
Social/racial inclusion quotas	−0.05	−0.16/0.06	0.355
Monthly family income	0.01	−0.11/0.13	0.892
Thoughts about student leave	−0.14	−0.29/−0.04	0.032
Academic semester	−0.02	−0.14/0.10	0.768
Moved home	0.09	−0.04/0.21	0.186
Social support	0.20	0.07/0.30	0.001
Sense of coherence	0.25	0.10/0.40	0.001
Anxiety	−0.22	−0.34/−0.10	< 0.001
Educational environment	−0.04	−0.14/0.06	0.469
Lifestyle	0.73	0.54/0.91	< 0.001
	**Lifestyle**		
	**β**	**95%CI**	**P**
Gender	−0.22	−0.38/−0.06	0.008
Monthly family income	0.04	−0.12/0.21	0.603
Academic semester	−0.05	−0.21/0.11	0.549
Moved home	−0.18	−0.34/−0.02	0.032
Social support	0.24	0.06/0.42	0.009
Sense of coherence	0.21	0.02/0.40	0.036
Anxiety	−0.21	−0.39/0.04	0.231
	**Educational environment**		
	**β**	**95%CI**	**P**
Gender	−0.16	−0.29/−0.03	0.013
Monthly family income	0.01	−0.12/0.14	0.910
Academic semester	0.05	−0.08/0.18	0.418
Social support	−0.02	−0.16/0.12	0.816
Sense of coherence	−0.13	−0.26/0.01	0.267
	**Anxiety**		
	**β**	**95%CI**	**P**
Academic semester	0.18	0.06/0.30	0.003
Moved home	0.04	−0.08/0.16	0.533
Social support	−0.24	−0.37/−0.12	< 0.001
	**Thoughts about student leave**		
	**β**	**95%CI**	**P**
Social support	0.13	−0.01/0.26	0.125
Anxiety	0.16	0.03/0.28	0.017
Educational environment	−0.03	−0.15/0.10	0.660
	**Social/racial inclusion quotas**		
	**β**	**95%CI**	**P**
Skin colour	0.27	0.16/0.39	< 0.001
Monthly family income	−0.05	−0.17/0.08	0.543
